# Ultrafast Laser Processing for High-Aspect-Ratio Structures

**DOI:** 10.3390/nano14171428

**Published:** 2024-08-31

**Authors:** Muyang Qin, Xinjing Zhao, Hanyue Fan, Ruizhe Leng, Yanhao Yu, Aiwu Li, Bingrong Gao

**Affiliations:** State Key Laboratory of Integrated Optoelectronics, College of Electronic Science and Engineering, Jilin University, Changchun 130012, China; qinmy1921@mails.jlu.edu.cn (M.Q.); zhaoxj126@163.com (X.Z.); fanhy1921@mails.jlu.edu.cn (H.F.); lengrz22@mails.jlu.edu.cn (R.L.); yanhao_yu@jlu.edu.cn (Y.Y.); liaw@jlul.edu.cn (A.L.)

**Keywords:** ultrafast laser processing, high aspect ratio, filamentation, Bessel beam, O-FIB

## Abstract

Over the past few decades, remarkable breakthroughs and progress have been achieved in ultrafast laser processing technology. Notably, the remarkable high-aspect-ratio processing capabilities of ultrafast lasers have garnered significant attention to meet the stringent performance and structural requirements of materials in specific applications. Consequently, high-aspect-ratio microstructure processing relying on nonlinear effects constitutes an indispensable aspect of this field. In the paper, we review the new features and physical mechanisms underlying ultrafast laser processing technology. It delves into the principles and research achievements of ultrafast laser-based high-aspect-ratio microstructure processing, with a particular emphasis on two pivotal technologies: filamentation processing and Bessel-like beam processing. Furthermore, the current challenges and future prospects for achieving both high precision and high aspect ratios simultaneously are discussed, aiming to provide insights and directions for the further advancement of high-aspect-ratio processing.

## 1. Introduction

Since the first attempt at ultrafast laser processing in 1987 [1], the capability of ultrafast lasers for material processing has garnered significant attention and achieved remarkable progress over the past few decades. Ultrafast lasers with pulse widths ranging from femtoseconds to tens of picoseconds exhibit extremely short pulse durations and exceptionally high peak intensities [2], making them promising for various applications such as laser ablation [3,4,5], three-dimensional (3D) structuring [6,7], drilling [8,9,10] and biomedical applications [11]. In the field of material processing, especially in tasks demanding high precision, efficiency, and flexibility, ultrafast lasers hold immense potential. Traditional long-pulse and continuous-wave lasers rely primarily on thermal damage mechanisms [12] for material processing, which are limited in achieving high precision due to the large heat-affected zone. In contrast, a significant advantage of ultrafast lasers is their ability to trigger nonlinear threshold effects [13] in materials, significantly improving processing resolution while significantly reducing the thermal diffusion area during laser processing [14,15,16]. Additionally, ultrafast laser processing exhibits numerous advantageous characteristics in the field of precision engineering. Among these, it boasts a remarkably wide range of material compatibility, encompassing diverse substrates and compositions. Moreover, the non-contact nature of ultrafast laser processing eliminates the potential material damage or contamination that may arise from physical contact. Crucially, the integration of ultrafast laser processing with various optical modulation devices [17] and tailored processing environments [18,19] significantly enhances its adaptability and flexibility, enabling it to meet increasingly complex and precise processing requirements and making it a valuable tool for applications ranging from microfabrication to surface modification.

In fact, the physical mechanisms underlying the interaction between ultrafast lasers and materials are complex, encompassing processes spanning temporal scales from femtoseconds to microseconds [20,21]. These include multiphoton absorption [22], avalanche ionization [23], electron-phonon scattering [24], and other phenomena. These mechanisms endow ultrafast lasers with unique processing effects. For instance, the nonlinear interaction between ultrafast lasers and materials occurs in a fleetingly short period [25], enabling rapid changes in the physical state of materials [26]. When combined with nonlinear threshold effects [27], multiphoton absorption concentrates laser energy near the focal point, enabling internal material processing and high-precision modification [28]. In terms of internal material processing [29], early research mainly focused on improving the processing resolution [30]. This entailed the utilization of high numerical aperture optics to induce localized damage in materials, including modifications [31], nanogratings [32], and nano-voids [33]. However, with the continuous advancement of nanotechnology and microelectronics science, the fabrication of many crucial micro-nano functional devices has evolved to demand not only superior lateral processing resolution but also substantial depth in the vertical dimension [34]. In particular, filamentation processing [35] and Bessel-like beam processing [36] have been introduced as two significant techniques, playing indispensable roles in improving vertical resolution and expanding applications [37,38,39]. Filamentation refers to the formation of a slender and high-energy laser filament [40] when an ultrafast laser propagates nonlinearly in a transparent medium, relying on mechanisms such as self-focusing induced by the Kerr effect [41], plasma-induced defocusing [42], dispersion [43], diffraction [44], and others [45,46,47]. This laser filament enables long-distance propagation within the medium while maintaining a high energy density, allowing high-precision processing of the material interior [48]. Bessel-like (including Bessel beam) beam processing, on the other hand, utilizes specific optical components or algorithms to transform the ultrafast laser beam into a Bessel-like beam, especially characterized by its unique properties such as non-diffracting, self-healing, and the preservation of beam focusing even during long-distance propagation [49]. The beam then exhibits certain characteristics similar to a Bessel beam, tailored precisely to meet the demands of practical applications [50,51]. These techniques often utilize a single pulse to fabricate high-aspect-ratio structures, serving as effective approaches for material processing [52].

In fields such as microelectronics, optics, and biomedical engineering, the production of high-aspect-ratio microstructures has always been challenging for many applications requiring high-aspect-ratio processing. For example, Zhao et al. [53] proposed a method for preparing microgrooves in fused silica, which requires high-aspect-ratio processing assisted by femtosecond Bessel beams. A laser modification region is formed by irradiating femtosecond Bessel beams, and then the modified region is removed by etching with hydrofluoric acid, resulting in uniform and straight microgrooves. F. Hendricks et al. [54] discussed the application of high-aspect-ratio processing in transparent media and proposed a new method to determine the optimal relationship between cutting width and scanning times for high-aspect-ratio machining. F. Courvoisier et al. [55] suggested that high-aspect-ratio processing is also required for nano drilling and curve direct laser processing, where the non-diffracting beam can alter the efficiency of high-speed material processing. Y. Zhang et al. [56] discussed the application of high-aspect-ratio processing in laser ablation and provided a simple and universal method for direct etching of materials without the use of chemical substances, which applies to the manufacture of inkjet nozzles. Huagang Liu et al. [57] reported the manufacture of crack-free microstructures using femtosecond laser-induced plasma-assisted ablation on sapphire substrates through laser heating.

Despite in-depth research efforts aimed at achieving high-aspect-ratio processing [58], researchers have encountered a significant impediment. It is always difficult to simultaneously achieve both high transverse precision and large-depth nanoprocessing, regardless of how the optical field modulation technology or specific processing parameters in ultrafast laser processing are improved [59]. Consequently, a delicate compromise must be made between precision and aspect ratio during the processing [60,61]. This predicament arises from the inherent nonlinear nature of the propagation of intense ultrafast pulses within transparent solids [62]. By tightly focusing the spot near the diffraction limit and pushing the processing parameters close to the material’s damage threshold, the transverse resolution of material processing can be maintained at the scale of hundreds of nanometers [63]. However, this approach simultaneously limits the longitudinal length to the order of tens of micrometers or even sub-micrometer scales. As a result, the challenge of controlling the longitudinal resolution persists in the realm of ultrafast laser aspect ratio processing.

In this paper, we review the fundamental characteristics of ultrafast laser processing, with a particular emphasis on the underlying principles and significant research related to the realm of high-aspect-ratio machining. Initially, we delve into the profound influence of the interaction between ultrafast lasers and materials on high-precision machining, highlighting the nonlinear effects such as threshold phenomena and multiphoton absorption. Following that, we elaborate on the principles of two key techniques, filamentation processing and Bessel-like beam processing, while emphasizing their indispensable role in the fabrication of intricate structures with exceptional aspect ratios. Furthermore, we present the current challenges encountered and promising future prospects. Our aim is to offer both theoretical insights and practical guidance for the continual evolution and refinement of ultrafast laser processing techniques in the domain of high-aspect-ratio machining.

## 2. The Multiphoton Absorption and Threshold Effect in Ultrafast Laser Processing

**Figure 1:**
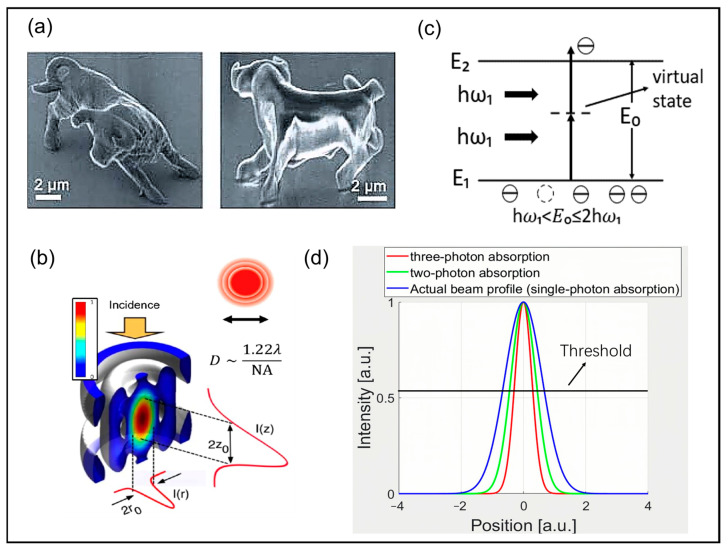
The principle of ultrafast laser processing. (**a**) Microbull sculptures produced by two-photon photopolymerization technology [68]; (**b**) Spatial intensity distribution of far-field focusing laser spots [69]; (**c**) Schematic diagram of two-photon absorption; (**d**) Schematic illustration of spatial distributions of laser energy intensity based on various absorption mechanisms: single-photon absorption (blue line), two-photon absorption (green line), and three-photon absorption (red line).

The unique feature of ultrafast laser processing lies in its capacity to penetrate objects without causing damage to the surface structure and to accurately perform processing within the internal area near the focus. This capability is attributed to the nonlinear effect induced by ultrafast lasers on transparent materials, which allows processing beyond the diffraction limit. The spatial resolution of laser processing is determined by the minimum resolution distance between two distinct features and is intricately linked to the diffraction limit. Defined by the diffraction limit, the size of the focused spot is contingent upon the numerical aperture *NA* and wavelength *λ*, as seen in Figure 1b. However, the ability to tightly focus the beam and achieve a very small spot size is influenced by the beam quality factor *M*^2^, which characterizes the divergence of the beam from an ideal Gaussian profile. In this scenario, the transverse dimension *d_f_* and the longitudinal dimension *z_f_* of the spot can be expressed by the formulas [7]:

(1) $$d_{f}\approx 2\frac{\lambda }{\pi }\frac{M^{2}}{NA},$$

(2) $$z_{f}\approx \frac{\lambda }{\pi }\frac{M^{2}}{{NA}^{2}},$$

where *d_f_* is the beam diameter at the focus, *z_f_* is the Rayleigh length and the factor $M^{2}=\frac{\pi \omega_{0}\theta }{\lambda }$. The numerical aperture given by NA=n×sin⁡θ, depends on the refractive index *n* and the aperture angle *θ* of the objective lens. Under the ideal focusing condition, the divergence angle of a Gaussian beam $\theta =\frac{\lambda }{\pi \omega_{0}}\;$ and thus $M^{2}=1$. Higher *M*^2^ values indicate beams with larger divergence angles, making it more challenging to achieve a small focused spot size for a given *NA* and λ. In specific processing applications, the actual spot size will vary due to the process flow. To enhance the resolution, using a high numerical aperture focusing objective lens is beneficial. However, increasing the numerical aperture leads to a decrease in working distance. In practical applications, a common approach to improve processing accuracy and reduce spot size is to utilize shorter wavelengths of light.

This process involves intricate interactions between laser and materials [25]. When a laser beam with an intensity that exceeds the material-specific and wavelength-dependent threshold initially hits the surface of a transparent material, multiphoton absorption occurs [22]. As accumulated photon energy reaches or surpasses the ionization potential of the atoms, multiphoton ionization ensues [64], prompting electrons to transition from bound states to free states and thus forming ions and free electrons. Subsequently, these high-energy free electrons collide with the surrounding bound electrons, triggering the ionization of the latter and the generation of additional free electrons, a phenomenon referred to as impact ionization [65]. The repetitive process of impact ionization gives rise to an exponential increase in the density of free electrons, producing a dense electron plasma within the material. This is the process known as avalanche ionization [66]. Then, energy is transferred to the lattice via electron-phonon coupling [67], creating localized regions of high temperature and pressure. Continuous input of laser energy results in the accumulation of thermal effects, diffusing within the transparent material and causing further heating. Eventually, this culminates in physical transformations such as melting, evaporation, and vaporization of the material.

In general, the extent of laser damage to materials depends on a complex interplay of physical and chemical reactions that occur after the material absorbs light. The threshold effect is a crucial factor, as the material will only sustain damage when certain parameters of the laser meet or surpass a specific threshold [70]. This threshold is tied to the optical properties of the material, the wavelength of the incoming light, the pulse width, and numerous other factors. As early as 1975, Wood, R. M. et al. [27] introduced various laser damage mechanisms and proposed a technique for measuring the laser damage threshold, which laid the groundwork for subsequent research. Additionally, in 1996, Hacker [71] suggested that laser pulses with a duration greater than 1 ns and a wavelength greater than 250 nm would result in laser damage to optical coatings. Then, in 1997, Glezer et al. expanded beyond surface damage [72] and demonstrated that using a pulse of 100 fs and 0.5 μJ tightly focused into the 200 μm region, they had successfully created cavities for the first time in fused silica, quartz, BK7 glass, sapphire, diamond, and acrylic by means of nonlinear absorption and micro explosions.

This is because when a laser is focused within a transparent material, either through an external lens or via internal self-focusing, the energy density typically fails to reach the damage threshold along the path of propagation before reaching the focal point. It effectively prevents unintended damage to the material. Only in the vicinity of the focus, a sharp increase in energy density can reach or exceed the damage threshold of the material, resulting in precise machining effects. In the process of laser damage to substances, the linear absorption process mainly occurs for narrow-band gap materials such as metals. It means that if the light intensity is not very large, the electron only needs to absorb a photon with an energy greater than the energy gap of the material to jump to a higher energy level. However, for materials with large bandgap widths, such as semiconductors or insulators, the situation is different. In these materials, multiphoton absorption is the main occurrence when a high-intensity laser is irradiated, as shown in Figure 1c. Here, an electron needs to absorb multiphoton with energies smaller than the energy gap simultaneously to jump from the valence band to the conduction band.

It is important to note that single-photon absorption typically takes place throughout the entire path of the laser as long as the photon’s energy is sufficient for the electron to be excited. On the other hand, multiphoton absorption predominantly occurs in the vicinity of the laser focus. This is because the energy density at the focal point can reach a high enough peak power to allow for the absorption of multiphoton simultaneously, facilitating the transition of electrons. Outside the focal point along the propagation path, the occurrence of multiphoton absorption is rare due to the lower energy density.

In optimal conditions, the intensity profile *I*(*r*) of the ultrafast laser beam typically exhibits a Gaussian distribution in space, described as:

(3) $$I\left(r\right)=I_{0}e^{\frac{-2r^{2}}{{\omega_{0}}^{2}}},$$

where *ω*_0_ is the beam waist radius, *r* is the actual spot radius, and *I*_0_ is the laser intensity. The effective absorption cross-section for *N*-photon absorption is proportional to the (*N −* 1)^th^ power of *I*(*r*) [69]. Although the actual focused spot size is generally determined by the focusing optics and the initial beam waist, multiphoton absorption preferentially takes place in regions of high intensity. Compared to single-photon absorption, the nonlinear characteristics allow highly concentrated local energy deposition under conditions where the laser energy density exceeds the material modification threshold, therefore surpassing the optical diffraction limit and promoting improved resolution. As seen in Figure 1d, single-photon absorption follows the linear intensity distribution. In contrast, multiphoton absorption necessitates higher light intensity, leading to a more concentrated spatial distribution of absorbed energy. Specifically, increasing the multiphoton order sharpens the energy absorption peak at the focus and accelerates the decline of absorption in the periphery [73].

When the laser energy is fine-tuned to approach the threshold, the material undergoes significant changes in the central region of the laser beam due to multiphoton absorption. These changes, which are influenced by the energy distribution characteristics of Gaussian-like beams, are confined to a very small spatial range. The threshold effect ensures that the material changes only in regions where the laser energy is sufficiently high. Therefore, by adjusting the laser energy close to the threshold, it is possible to achieve a spatial resolution that surpasses the diffraction limit [74]. One remarkable example of this is a study conducted in 2002 by Tanaka, T., H.-B. Sun, and S. Kawata [26], where they utilized two-photon polymerization technology with multiphoton absorption to precisely control laser energy to approach the threshold for two-photon polymerization. This resulted in the fabrication of a nanobull with a three-dimensional structure (Figure 1a). In 2005 [30], they further improved the lateral resolution from 120 nm to 100 nm, marking a significant advancement in laser processing for enhancing accuracy. This progress not only contributes to the greater understanding and utilization of the multiphoton effect of ultrafine lasers but also holds promise for creating valuable models.

Ultrafast laser processing utilizes nonlinear effects like multiphoton absorption and threshold effect to accurately focus high-energy-density laser pulses inside transparent materials, allowing for precise internal modification processing [63]. When combined with a mobile platform, this method enables precise control over the location of the laser focus, facilitating high-quality machining in various areas within the material. This approach not only offers high precision and efficiency but also helps avoid surface damage that can occur with traditional processing methods, leading to groundbreaking changes in material processing.

The nonlinear threshold effect exhibits certain instability in practical applications. This is due to the difficulty in ensuring a completely constant output power of the actual laser. When the provided energy approaches the energy threshold [75], even the slightest disturbance can significantly amplify the nonlinear effect. Slight attenuation of the laser pulse may render the material completely undamaged, while minor enhancement may lead to rapid expansion of the damaged area. To overcome this challenge, further in-depth research and understanding of the nonlinear threshold effect is necessary to optimize its application methods for achieving higher resolution and stability in ultrafast laser processing technology [76].

## 3. Filamentation Processing

Initially, the self-focusing effect was first proposed in the 1960s [77], and the filamentary damage induced by ultrashort pulse lasers in glass was discovered for the first time [35]. Since then, the phenomenon of laser filamentation has emerged as a research hotspot in the fields of optics and material processing. This phenomenon has been extensively and thoroughly investigated under various medium conditions, such as air and transparent solids [78,79,80], demonstrating promising applications in areas like supercontinuum generation [81], atmospheric propagation [82], optical fiber [83] and cutting [84]. Critically, filamentation based on ultrafast laser processing not only reveals unique physical mechanisms but also brings further breakthroughs in the fabrication of structures with high aspect ratios.

By focusing through specialized devices, ultrafast lasers converge into beams with exceptionally high peak power densities, resulting in a lower intensity at the beam center compared to its edges. This intensity gradient triggers a significant change in the refractive index of the central region, where light propagates slower than at the periphery. In essence, the formation of nonlinear laser filaments stems from the dynamic equilibrium between self-focusing, induced by the Kerr effect, and self-defocusing caused by plasma generation [85]. The nonlinear transport equation commonly used to describe the temporal and spatial dynamics of ultrafast laser filamentation is given by [86]:

(4) $$2ik_{0}\frac{\partial A}{\partial z}+\left(1-\frac{i}{\omega_{0}}\frac{\partial }{\partial \tau }\right){∆}_{⊥}A+2k_{0}DA+\mu_{0}{\omega_{0}}^{2}\left(1+\frac{i}{\omega_{0}}\frac{\partial }{\partial \tau }\right)p_{NL}=0,$$

where *A(z,r,t)* represents the spatiotemporal envelope of the laser pulse, *ω*_0_ is the central frequency, *k*_0_ is the linear propagation constant, *μ*_0_ is the permeability of free space, ∆_⊥_ is the Laplacian operator in the xy plane, *D* describes the high-order dispersion, and *p_NL_* denotes the nonlinear polarization. This series of parameters encapsulate the interplay of multiple optical effects, including spatiotemporal coupling, diffraction, nonlinear refraction, group velocity dispersion, and self-steepening, that underpin the filamentation process.

**Figure 2:**
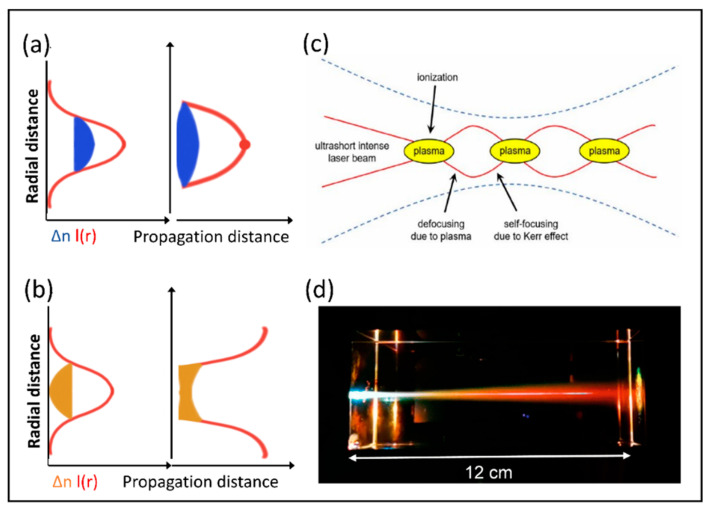
The principle of filamentation. (**a**) Laser intensity and refractive index in the case of self-focusing [88]; (**b**) Laser intensity and refractive index in the case of defocusing [88]; (**c**) The focusing–defocusing cycles undergone by the intense core of the beam during the formation of the filament [88]; (**d**) Physical scene of femtosecond laser filamentation in a solid medium [86].

During the nonlinear propagation of femtosecond laser in space, when the incident laser peak power Pin exceeds the critical power *P_cr_*, the nonlinear Kerr effect dominates, leading to self-focusing of the beam. Conversely, if *P_in_* is below *P_cr_*, the beam primarily undergoes diffraction. The critical threshold power is defined as:

(5) $$P_{cr}=\frac{3.72\lambda_{0}^{2}}{8\pi n_{0}n_{2}},\;$$

where *λ*_0_ is the laser wavelength in vacuum, *n*_0_ is the refraction index of the medium in vacuum, and *n*_2_ is the nonlinear Kerr index. This self-focusing phenomenon leads to a significant increase in the refractive index at the beam’s center, effectively overcoming beam diffraction and further enhancing the power density. When the optical intensity reaches a threshold sufficient to ionize air molecules, it induces the formation of air plasma. These plasmas, characterized by a lower refractive index, subsequently cause the beam to defocus, resulting in the propagation and expansion of an elongated plasma channel in the air. Figure 2a,b visually depict the variations in refractive index and optical intensity during the processes of self-focusing and self-defocusing. Self-focusing, stemming from the Kerr effect, manifests as a positive exponential change in refractive index, accompanied by a relatively high refractive index. At this stage, the medium’s refractive index behaves akin to a focusing lens, converging parallel beams and maximizing optical intensity at the central focus. However, the formation of abundant plasmas leads to a negative refractive index with a lower value, functioning equivalently to a diverging lens that defocuses the beam [87]. It is worth noting that the defocused beam may still retain sufficient power to reinitiate self-focusing, thus establishing a cyclic process of self-focusing and self-defocusing. This cyclic mechanism lies at the heart of ultrafast laser filamentation, with filament length determined by the distance over which these cycles repeat. Figure 2c illustrates the cyclic focusing and defocusing experienced by the intense core of the beam during the filamentation process [88]. The solid line represents the diameter of the intense core, which decreases with self-focusing and increases with self-defocusing. The surrounding dashed line denotes the root-mean-square radius of the entire beam, providing a reference for understanding the overall morphology of the filamentation process. This process, which collectively shapes the phenomenon of filamentation, showcases the dynamic competition between the positive refractive index changes induced by the Kerr effect and the negative refractive index changes induced by plasmas.

In his seminal work, Marburger first provided a precise description of the self-focusing propagation distance *Lc* of the beam [89]:

(6) $$L_{c}=\frac{0.367L_{DF}}{\sqrt{{\left(\sqrt{\frac{P_{in}}{P_{cr}}}-0.852\right)}^{2}-0.0219}},$$

where the Rayleigh length of the beam, *L_DF_*, a crucial parameter quantifying the diffraction-spreading characteristics, is defined as $L_{DF}=\frac{{{\pi \omega }_{0}}^{2}}{\lambda }$,with *ω*_0_ being the beam waist and *λ* the laser wavelength in the medium. This expression conspicuously reveals the fact that the length of self-focusing is directly correlated with the squared diameter of the beam. The slender filaments formed by ultrafast lasers efficiently confine laser pulse energy within a minute spatial domain, permitting the laser to traverse extensive distances in transparent media without notable divergence. This propagation characteristic significantly surpasses the limitations imposed by the Rayleigh length of conventional beams, highlighting the unique advantages of ultrafast laser filamentation in energy transmission. As the laser pulse propagates through the medium, the formed plasma channel gradually relaxes after modifying the material. Figure 2d presents a vivid illustration of the actual filamentation process induced by a femtosecond laser in a solid medium.

**Figure 3:**
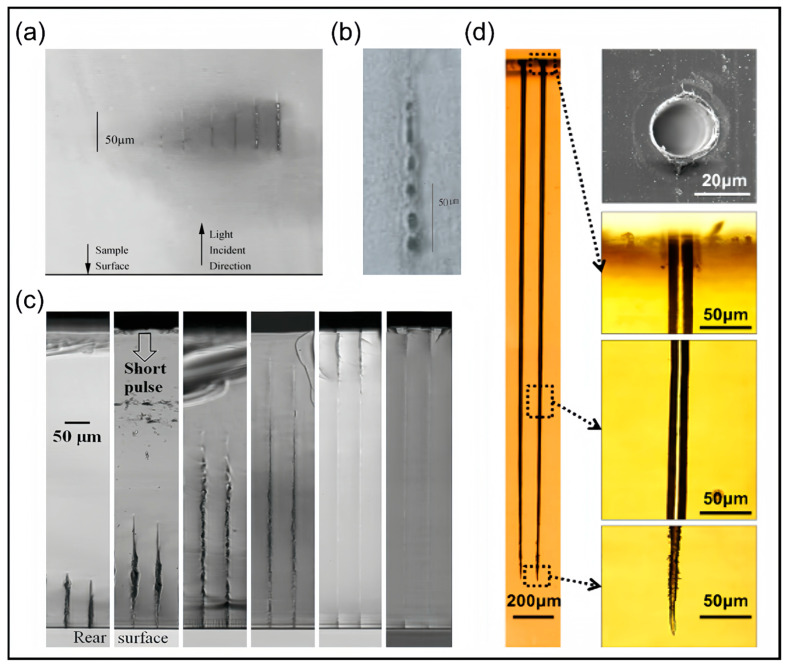
The filament induced modification and ablation. (**a**) The filament structure in fused silica induced by light intensity originates proximate to the laser’s geometric focus and extends along the laser pulse’s propagation [90]; (**b**) A side-view illustration showcasing a series of voids within a fused silica filament resulting from the application of a 250-fs laser pulse with a pulse energy of 80 microjoules [90]; (**c**) Enhancing the thickness of the soda-lime glass plate placed behind the objective lens will hinder the radial propagation of the pulse and amplify the power density at the focal point [92]; (**d**) Subjected to identical laser parameters, the depth of processing achieved in a vacuum environment is twice as deep compared to that in air [93].

In the experimental realm, numerous researchers have employed ultrafast lasers to process transparent materials, delving into the performance characteristics of filamentation effects. For instance, Luo et al. [90] induced filamentary structures in fused silica using varied light intensities, as exemplified in Figure 3a, therefore validating several theoretical predictions of the filamentation phenomenon. Their findings revealed that filament formation initiates at the laser focus and subsequently elongates significantly along the laser propagation direction, with the longitudinal length enhancing from 15 μm to 50 μm before reaching saturation. In further experiments, they generated a series of voids in fused silica using a 250 fs, 80 μJ laser pulse, as shown in Figure 3b. The result visually showcases the ultrafast laser refocusing phenomenon during filamentation, elucidating the dynamic modulation of the laser focus by the nonlinear refractive index changes in the medium, giving rise to several voids at distinct locations. However, when switching to BK7 optical glass and LiNbO_3_ crystals, although filament formation and elongation remained similar, the subsequent void series was not observed. This is likely attributed to the lower nonlinear refractive index, which prevented sufficient energy concentration during laser propagation and, consequently, failed to trigger the refocusing effect. Sun et al. [91] utilized a conical lens to form filaments and reported a linear increase in filament length with laser energy, further enriching our understanding of the filamentation mechanism. These experimental investigations and theoretical discussions during processing not only deepened our comprehension of the filamentation mechanism but also propelled innovations and advancements in ultrafast laser processing technologies.

Several studies have proven that exploiting nonlinear, elongated filamentary damage in ultrafast laser processing serves as an effective approach to achieving nanostructures with high aspect ratios. At the same time, researchers have also presented a series of methods to enhance the aspect ratios of processed microstructures. Bhuyan et al. [94] employed conical lens-generated filaments to create non-tapered microchannels with a diameter of 2 μm and a depth of 83 μm in borosilicate glass samples, showcasing the potential of filamentation processing in precision manufacturing. Furthermore, Ahmed et al. [92] observed single-hair filamentary voids with a diameter of 3.8 μm and a length of 700 μm in glass, yielding a high aspect ratio of 184. They highlighted that by controlling the thickness of a soda-lime glass plate placed behind the objective lens while maintaining constant laser power, one can regulate the width and length of the filaments. As depicted in Figure 3c, an increase in glass plate thickness leads to tighter laser focusing, hindering radial pulse propagation and enhancing power density at the focus, resulting in longer, narrower, and more stable filaments. Additionally, Xia et al. [93] demonstrated an aspect ratio exceeding 100 in vacuum conditions, as seen in Figure 3d. They compared the aspect ratios of channels processed in air and vacuum and found that, under identical laser parameters, while the diameters remained nearly constant, the depths achieved in vacuum were twice those in air. This discovery underscores the significant influence of processing ambient pressure on micropore morphology, as well as revealing the energy loss mechanisms of plasma in gas molecules.

**Figure 4:**
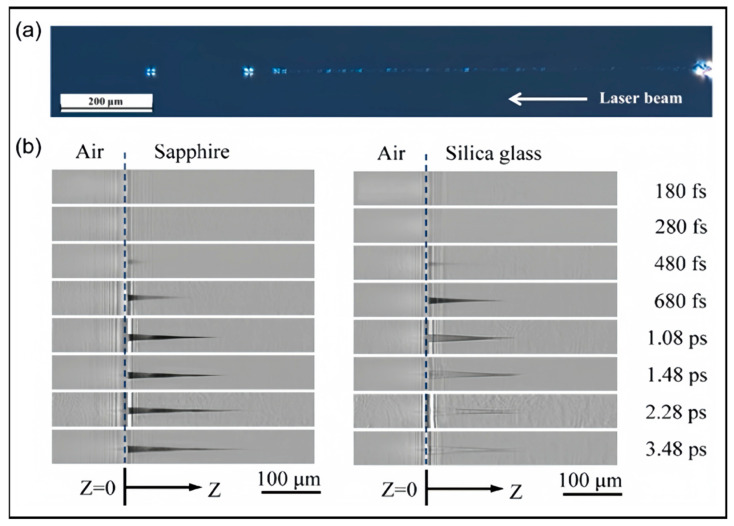
The ultrafast evolution of filaments. (**a**) An image showcases the extended filamentary track length (1180 μm) achieved within sapphire with 107 µJ pulse energy, 300 kHz repetition rate, and 10,000 pulses [95]; (**b**) A series of depictions of the evolution of filaments within sapphire and silica glass on different femtosecond and picosecond timescales with pump energy of 50 µJ and a relative position D = 60 µm [98].

Recently, researchers have focused on optimizing processing parameters for filamentation to cater to practical applications. Amina’s team systematically investigated the effects of pulse energy, repetition rate, and pulse count on filamentary characteristics in sapphire, a material with a wide bandgap and high hardness [95]. They found that pulse energy primarily governs laser filament growth, while pulse count induces transverse and longitudinal crack damage, and repetition rate determines the distance from the damage initiation point to the sample surface. Under experimental conditions of 107 μJ pulse energy, 300 kHz repetition rate, and 10,000 pulses, Figure 4a shows a filamentary trace, which is successfully observed with a length of 1180 μm, providing valuable insights for precision sapphire processing. Similarly investigating laser filamentation traces in sapphire, Xu et al. paid more attention to the focal depth as the key processing parameter [96]. Varying the focal depth from 0 to 60 μm at a single pulse energy of 9.445 μJ, they utilized their developed femtosecond laser point-to-point filamentation technique and achieved filamentary traces in sapphire fibers. Optimal results were obtained at a focal depth of 10 μm, yielding a high-quality filament with a maximum length of 90 μm. This effective utilization of filamentation technology supports the fabrication of high-quality sapphire fiber Bragg gratings, fostering applications in high-temperature sensors for aerospace and chemical industries. To cater to the demands of film cooling in aerospace engine applications, Liu et al. have devoted their efforts to refining high-aspect-ratio microholes in high-temperature resistant SiC composites [97]. Based on the spatial energy density distribution stemming from the femtosecond filamentation effect, they primarily adjusted the negative defocusing distance as a key parameter. In the experiment, they varied the negative defocusing distance from 0 to −7 mm and the observed result is a trend where the hole depth initially increases and then decreases. Notably, at defocusing distances of −3 mm and −4 mm, through-holes are achieved with maximum hole depths, optimal ablation efficiency, the best circularity at the exit, and minimal tapering off the sidewall. This underscores the efficacy of adjusting the defocusing distance in enhancing the morphology and performance of drilled holes. Moreover, Ren et al. employed time-resolved shadowgraphy to investigate filament evolution within sapphire and silica glass and discussed the effect of plasma on the filament length [98], therefore validating the crucial role of pulse energy and focal depth in influencing filament length, as previously argued. Figure 4b clearly showcases the comparison of filamentation trajectories in sapphire and silica glass at diverse femtosecond and picosecond scales, which is in favor of the application of laser filamentation in precision processing.

Relying on its advantages as highly concentrated beam energy and unique self-focusing and defocusing mechanisms, laser filamentation enables the fabrication of elongated fine structures whose lengths significantly exceed their diameters. In essence, research on filamentation processing has unveiled novel avenues and presented developmental opportunities for achieving high-aspect-ratio processing using ultrafast laser technology. Furthermore, through a series of innovative methodologies and strategies, it has significantly bolstered processing precision and efficiency, ushering in revolutionary advancements in the realm of micromanufacturing. While acknowledging the unique advantages of ultrafast laser filamentation technology in nanomanufacturing, it is important to consider its inherent limitations. First, material compatibility is a constraint, as this technology primarily excels with transparent materials and encounters challenges with non-transparent or strongly absorbing materials. For instance, materials with lower nonlinear refractive indices, such as BK7 optical glass and LiNbO_3_ crystals, may fail to effectively trigger filamentation under certain conditions. Second, the instability and unpredictability of nonlinear effects pose a significant challenge. The filamentation process demands rigorous control over the uniformity of laser pulse intensity, the stability of beam propagation in the medium, and the regulation of plasma defocusing. Slight variations in parameters can have a significant impact on the filamentation process, leading to uneven filament structures, disruptions, or inability to extend to the desired length. Hence, when harnessing the filamentation phenomenon for high-precision processing, we need to comprehensively consider its limitations and applicable scopes to ensure that it exerts its maximum advantages with appropriate materials and conditions, as a result fulfilling more various processing requirements.

To overcome these hurdles with the advancement of ultrafast laser technology, researchers are delving deeper into the intrinsic laws governing filamentation mechanisms and optimizing processing parameters to achieve more predictable and controllable nonlinear effects during the filamentation process [99,100]. Additionally, exploring diverse optical field manipulation techniques, such as Bessel-like beam processing, holds promise for enhancing processing precision and efficiency. For instance, in scenarios requiring the suppression of detrimental nonlinear effects [101], specialized fiber gratings and temporal-spatial focusing techniques can be utilized for beam shaping to fulfill practical processing demands [102].

## 4. Bessel Beam Processing

In 1987, Durnin first described the quasi-non-diffracting Bessel beams, a discovery that brought a major breakthrough in the field of optics [103]. He proved that Bessel beams are non-diffracting solutions to the Helmholtz equation. This property allows Bessel beams to maintain their transverse intensity distribution without significant diffraction over propagation distance. From a physical perspective, the intensity distribution of Bessel beams exhibits a unique concentric ring structure in the transverse plane. A high-intensity bright spot is at the center, surrounded by a series of coaxial rings with gradually decreasing brightness. This structure can be viewed as the interference pattern formed by multiple plane waves converging at the focus. Each plane wave contributes a portion of its energy to the overall beam distribution.

**Figure 5:**
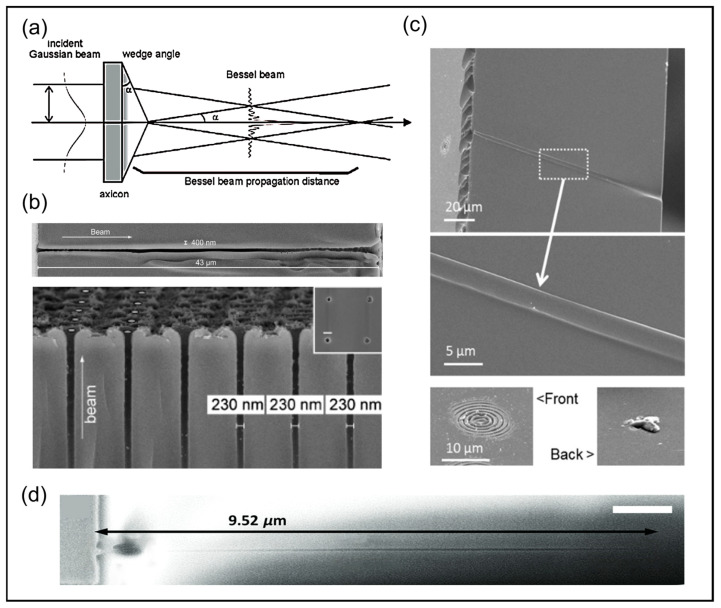
(**a**) The demonstration of using an axicon illuminated with a Gaussian beam to create a Bessel beam [17]; (**b**) The above SEM image depicts a through channel in glass with an aspect ratio approaching 100, achieved using a single shot from a Bessel beam, while the bottom SEM image displays an array of channels with each channel having a length of 10 μm, a diameter of 230 nm, and a pitch of 1.6 μm [105]; (**c**) The SEM images, respectively, represent a channel cut and structures at the top and back surfaces of the microchannels formed with single pulses [106]; (**d**) The SEM image of the nanochannel structure when the sample surface is put 0.9 μm above the focal plane of the objective lens by a single-shot femtosecond Bessel beam with 1.0 μJ [107].

The common method for generating Bessel beams is by passing a Gaussian beam through a conical lens, as shown in Figure 5a. When a converging lens is placed behind the conical lens, a ring-shaped beam is produced at the focal plane of the lens. The radial intensity distribution of the Bessel beam formed by the cone axis can be approximated as [104]:

(7) $$\frac{I(\rho ,z)}{I_{0}}=\lambda k_{⊥}^{2}zJ_{0}^{2}\left(k_{⊥}\rho \right),$$

where *k*_⊥_ is the transverse component of the wave vector, and *J*_0_ is the zeroth-order Bessel function. For a standard Bessel–Gaussian beam, the cone angle determines the degree of transverse divergence, i.e., the width of the main lobe and the size of the side lobes. The initial Gaussian beam waist determines the length of the diffraction-free propagation region. Bessel beams have some distinct properties compared to traditional Gaussian beams. A characteristic of Gaussian beams is that the intensity is maximum at the center and gradually decays towards the edges. Although a focused Gaussian beam can maintain a high energy density for a short time, the transverse spreading of energy limits the longitudinal propagation distance. As the propagation distance increases, its intensity decays rapidly. In contrast, Bessel beams exhibit a central core and a ring structure, enabling a more concentrated and powerful light spot. At the same time, they demonstrate a longer depth of focus, with the intensity remaining relatively stable even at positions far from the focal point. During propagation, although the radial dimensions of the central lobe are similar for both beams, Bessel beams have a much larger longitudinal propagation distance.

Bessel beams have attracted attention due to their properties of diffraction-free propagation, self-reconstruction, and long depth of focus. During propagation, this type of beam can maintain a stable shape and intensity distribution, experiencing almost no noticeable diffraction. Owing to its special phase distribution, when encountering an obstacle, the main lobe of a Bessel beam can self-reconstruct and continue propagating in its original form. The nonlinear propagation of Bessel beams allows for the generation of uniform plasma filaments, reducing the spatiotemporal distortion of light propagating through materials. These outstanding performances have demonstrated the immense application potential of Bessel beams in fields such as optical communications [108], high-resolution optical imaging [109], advanced materials processing [110,111], and nanolithography [112].

As an important indicator of a beam’s ability to maintain its shape and energy density, the depth of focus is typically measured by the Rayleigh range. In traditional laser processing, material modification is often subject to the focusing limitations associated with diffraction. However, due to its diffraction-free nature, Bessel beams can avoid the focusing limitations caused by diffraction effects during processing, overcoming the diffraction limit and enabling the realization of structural features that surpass optical resolution limits [113]. Since the central light spot diameter in a Bessel beam is extremely small, and its energy can be transmitted over long distances while remaining essentially unchanged, these superior properties make it suitable for processing high-aspect-ratio structures, expanding the possibilities of laser-material processing. It is worth mentioning that Bessel beams are primarily applicable for processing transparent materials. For opaque materials, the conical energy flux generated at the main lobe cannot propagate.

In the continuous exploration of ultrafast laser processing technology, the first report of successful optical damage using an ultrashort pulsed Bessel beam was published in 2001 [104]. This pioneering result revealed the potential of Bessel beams in the field of ultrafast laser-material processing, particularly for the efficient fabrication of high-aspect-ratio structures. Since then, research in this area has rapidly gained momentum, witnessing a gradual increase in the achievable aspect ratios utilizing ultrafast Bessel beam techniques. Specifically, as shown in Figure 5b, a single femtosecond Bessel beam successfully fabricated through-holes with a diameter of 43 μm and a depth of 400 nm, as well as channel arrays with a width of 230 nm and a spacing of 1.6 μm in glass, representing a significant advancement in femtosecond laser micro/nanofabrication [105]. Furthermore, Xie precisely controlled the local electron density of a femtosecond laser through wavefront shaping and achieved microholes with an aspect ratio as high as 330 in polymethyl methacrylate using a single femtosecond Bessel beam [114]. In 2014, Bhuyan et al. pushed the capabilities of ultrafast Bessel laser processing to new heights, realizing micro/nanostructures with aspect ratios exceeding 10^3^ [115]. Research by Mitra et al. also demonstrated the superior performance of ultrafast Bessel processing. Unlike previous works, Mitra et al. emphasized the advantage of high-energy beams, reporting the formation of microchannels with an aspect ratio up to 1200 in borosilicate glass using a single 1 mJ high-energy Bessel pulse [106]. Figure 5c shows a scanning electron microscope image detailing a cross-sectioned microchannel formed in their experiment, including the precise correspondence between the top incident beam cross-section and the bottom ablation features, providing direct evidence of the high-aspect-ratio through-hole drilling. Recently, Yu et al. [107] achieved nanometer-scale precision in this field. By optimizing the energy deposition strategy, they realized efficient simultaneous surface and bulk modification of the sample using a single femtosecond laser pulse, resulting in nanochannels with a minimum feature size of 18 nm and an aspect ratio exceeding 200, as shown in Figure 5d. In-depth research on Bessel beam processing technology has not only greatly advanced the field of ultrafast laser processing but also provided powerful technological support and promising application prospects for the fabrication of high-aspect-ratio micro/nanostructures, indicating that this field will continue to lead the innovation and breakthroughs in micro/nanomanufacturing in the future.

While acknowledging the unique advantages of Bessel beam processing, we cannot overlook its existing drawbacks. First, the microstructures fabricated exhibit a conical shape, which, to some extent, limits their application in situations requiring strict control over the structural shape. Second, Bessel beams have limited depth of focus and energy. When the processing depth exceeds the depth of focus range, the beam will gradually lose its focused state, leading to a decrease in energy density and deterioration in processing quality. Side lobe ablation is also a major challenge in practical applications [116,117]. The side lobes carry a portion of the energy, which in some cases may cause undesired ablation. The ablated products accumulate in the processing area, degrading the quality of the microchannels. Inevitably, to achieve deeper structures, the processing slot width needs to be increased to compensate for the depth limitation. This leads to increased material loss and reduced processing efficiency. Therefore, in practical applications, to amplify specific advantages and avoid certain drawbacks, we often need to shape the Bessel beam, tailoring its performance to suit the requirements of specific scenarios.

**Figure 6:**
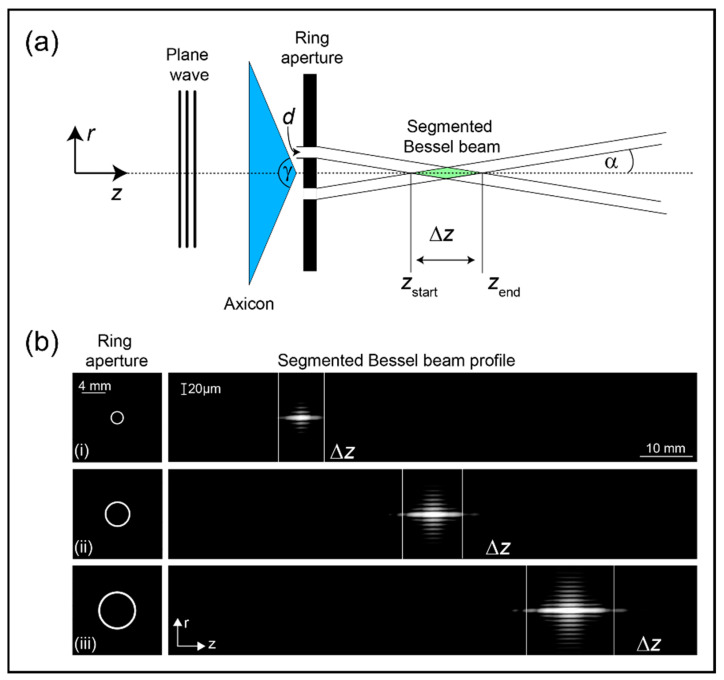
The generation and measurement of segmented Bessel beams [122]. (**a**) A schematic diagram of the segmented Bessel beam region ∆z generated by placing a ring aperture with aperture size d behind an axicon with apex angle γ; (**b**) With an axicon angle γ = 170◦ and wavelength λ = 632.8 nm, the illustration depicting the position and length of the segmented Bessel beam with different optimal apertures *d*_min_ and radial positions: (i) *r* = 1 mm, *d*_min_ = 197.85 µm, (ii) *r* = 2 mm, *d*_min_ = 281.58 µm and (iii) *r* = 3 mm, *d*_min_ = 344.90 µm.

To generate Bessel beams in early explorations, people used a single annular slit or aperture [118]. Although simple in structure, this method may block most of the incident radiation, resulting in low energy utilization efficiency and unsuitability for most processing scenarios. Subsequently, the widely used axicon [119], as a more general solution, often requires cooperation with other optical devices such as spatial filters [120,121]. Currently, the modulation method combining axicon lenses with masks has become a simple and efficient technique. It first generates a Bessel beam using an axicon lens and then uses a mask for fine-tuning. For example, Mulle et al. used a replaceable annular aperture placed behind the axicon lens to flexibly produce segmented Bessel beams, as illustrated in the schematic diagram shown in Figure 6a [122]. Their concept was validated in Figure 6b, where it was evident that manipulating the radial position and size of the annular aperture can skillfully control the generation position and length of the segmented Bessel beams. This study not only enriches the modulation strategies for Bessel beams but also provides substantial support for subsequent applications of Bessel beam processing. To realize the objective of precise control over the ablation depth during dielectric surface processing, researchers introduced the annular filtering aperture truncated Gaussian-Bessel beam technique to regulate the focusing depth, therefore significantly enhancing processing precision [123]. Relying on the truncated Gaussian-Bessel beam, researchers not only successfully fabricated a 10 × 10 dense microchannel matrix array with different spatial pitches but also verified the accuracy of the spacing between the cavities through Energy Dispersive Spectroscopy-assisted post-processing polishing sample surface scanning electron microscopy images. To fabricate pore arrays, Liu et al. combined an axicon and an amplitude mask, specifically an annular aperture, resulting in a suitably truncated micro-Bessel beam with adjustable lengths [34]. This method effectively prepared periodic nanopore arrays with adjustable depths, significantly benefiting the manufacturing of advanced optical devices. They visually reveal the morphology of single cavities and nanopore arrays with spacings of 3 μm and 1.5 μm at laser pulse energies of 900 nJ and 650 nJ, respectively. This fully demonstrates the critical role of laser pulse energy and spatial period as key parameters in controlling the machining depth of channels. 

**Figure 7:**
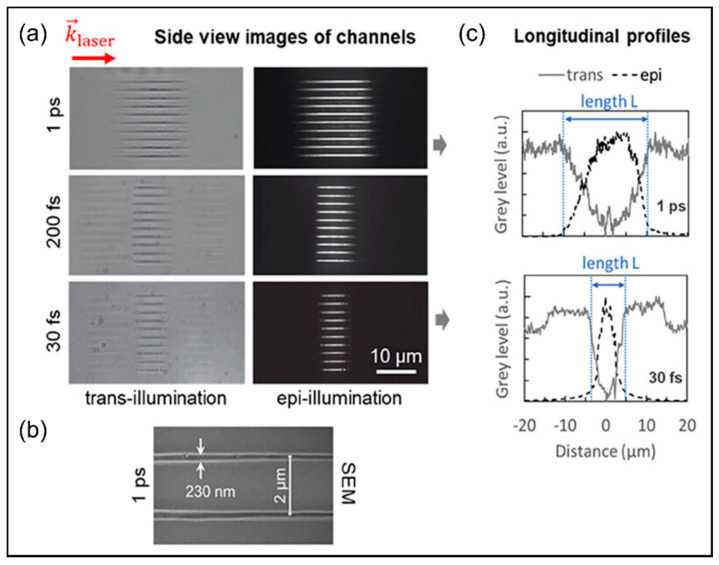
Analysis of the nanochannel characteristics generated by the skillfully shaped Bessel beam [124]. (**a**) Side-view trans-illumination images and epi-illumination images of a series of 10 channels in fused silica, fabricated by single-shot ablation with the energy ≈ 0.5 μJ and pulse duration of, respectively, 1 ps, 200 fs, 30 fs; (**b**) An SEM image of channels is also shown for the case 1 ps; (**c**) Longitudinal profiles of a channel extracted from images for series 1 ps and 30 fs.

Building on previous research advancements, recent considerable efforts by Datta et al. aimed to transcend the limitations of planar nanolithography by focusing on three-dimensional integrated processing beam shaping [124]. They designed a sophisticated experimental setup to customize the Bessel beam by combining an axicon and an annular aperture to generate a truncated Bessel beam, which was further demagnified using a lens and a microscope objective. As shown in Figure 7a, by processing fused silica with 1 ps, 200 fs, and 30 fs laser pulses, the research team successfully obtained a series of 10 channels using both trans-illumination and epi-illumination microscope modes. The SEM image in Figure 7b captures the channel details at 1 ps, revealing a spacing of 2 μm between each channel. Furthermore, Figure 7c directly compares the longitudinal profiles of 1 ps and 30 fs pulses under both microscopy modes, clearly demonstrating the significant advantage of 1 ps pulses in forming longer and more uniform nanochannels compared to shorter pulse durations. At the forefront of current technological exploration, the ingenious collaboration between axicons and masking techniques has emerged as a potent tool for modulating Bessel beams, leading to the dense integration of nanochannels with adjustable lengths. This advancement holds promise for propelling the vibrant development of 3D nanomanufacturing and chip lithography.

It should be noted that in practical processing applications, not only is the Bessel beam used, but also the Bessel-like beam. An ideal Bessel beam theoretically carries infinite energy, which is impossible to achieve in practice. However, within a limited spatial range, we can employ certain methods to obtain Bessel-like beams with very similar properties. For instance, as a highly integrated and flexible programmable digital device, the spatial light modulator can adjust the beam shape and parameters in real time [125,126,127]. However, its lower energy threshold limitation and high cost restrict its application in certain fields. Bessel-like beams not only inherit the key fundamental characteristics of Bessel beams, such as spin angular momentum and non-diffraction but also are improved or extended through innovative optical designs and processing techniques, potentially generating or optimizing new properties. Therefore, these advantages enrich the technological options in the processing field and demonstrate high application value.

Kong et al. conducted a detailed comparative analysis of several aspects between Bessel beams and Bessel-like beams in their work [128]. Due to the intensity distribution of the Bessel beam can be mathematically expressed as follows [129]:

(8) $$I_{BB}\left(r,z\right)=2\pi kI_{0}\beta^{2}ze^{-2{\left(\frac{z\beta }{\omega }\right)}^{2}}J_{0}^{2}\left(k\beta r\right),$$

where *k* represents wave number, *I*_0_ represents the peak intensity of the Gaussian beam, *ω* represents the waist radius of the Gaussian beam, *α* represents the bottom angle of the axicon, and β ≈ (n−1) α, *n* represents the refraction index of silica glass for *λ* = 1030 nm. Through a series of derivations, the team derived the intensity distribution of the Bessel-like beam as:

(9) $$I_{BLB}\left(r,z\right)=2\pi k{I_{0}(\frac{\beta }{\frac{z}{f}+1})}^{2}[(\frac{z}{\frac{z}{f}+1})e^{-2{\left(\frac{z}{\frac{z}{f}+1}\right)}^{2}\times {\left(\frac{\beta }{\omega }\right)}^{2}}J_{0}^{2}\left(k(\frac{\beta }{\frac{z}{f}+1})r\right)]$$

**Figure 8:**
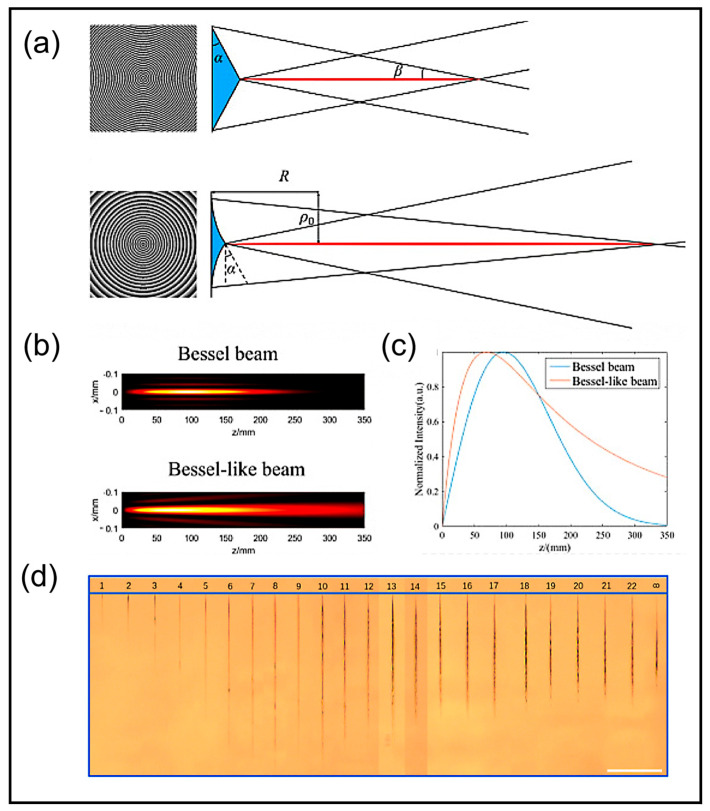
Comparisons between Bessel beams and Bessel-like beams [128]. (**a**) Theoretical holograms of the axicon to create Bessel beam and the simplified arc axicon to create Bessel-like beam; (**b**) Intensity distribution of the Bessel beam and Bessel-like beam; (**c**) On-axis intensity distribution of the Bessel beam and Bessel-like beam; (**d**) Variation in ablation length with different Bessel-like beams (*k* = 1–22) and Bessel beam (*k* = ∞).

It is proposed that the characteristics can be determined by *ρ*_0_/*ω*, where *ρ*_0_ represents the distance between the point of tangency and the optical axis. In their experiments, the researchers employed a spatial light modulator loaded with a simplified phase pattern of an arc axicon to generate the Bessel-like beam, which exhibited advantages over the Bessel beam, including slower on-axis intensity variation, tunable non-diffracting length and enhanced efficiency in ablating microholes. Figure 8a presents holograms, one for generating a Bessel beam using an axicon and another for generating a Bessel-like beam utilizing a simplified arc axicon. Moreover, Figure 8b,c contrast the intensity distributions and on-axis intensity distributions of the Bessel beam and Bessel-like beam, respectively, highlighting the longer non-diffracting propagation length and slower on-axis intensity variation of the Bessel-like beam. Furthermore, in order to explore optimized parameters for Bessel-like beams, ablation experiments on silica glass were conducted using beams with varying *ρ*_0_/*ω*, where *k* was used as a proxy for *ρ*_0_/*ω* due to the relationship $\frac{\rho_{0}}{\omega }=2^{0.25\left(k-1\right)-1}$. Figure 8d shows the ablation length variation for *k* = 1–22 and *k* = ∞, concluding that the optimal range for Bessel-like beams lies within *k* = 6–11(*ρ*_0_/*ω* = 1.18–2.83). The study clarifies the similarities and differences in intensity distributions between the Bessel beam and Bessel-like beam through precise mathematical derivations and experimentally determines the optimal operating range of Bessel-like beams under specific conditions. This comprehensive comparison of Bessel and Bessel-like beams across multiple dimensions provides crucial insights for the further development of beam shaping techniques and precise applications with tunable processing depths. Several researchers have innovatively pursued the potential of Bessel-like beams in precision processing, aiming to overcome the limitations of conventional Bessel beams. For instance, Wu et al. [130] devised a telescopic system to generate Bessel-like beams, retaining the compact main lobe diameter and high density of Bessel beams in the cross-section. This system is expected to excel in fabricating high-aspect-ratio subwavelength structures on the curved surfaces of Diamond-ZnS composite materials. To overcome the limitation of traditional Bessel beams with poor non-diffractive length adjustability, Yao et al. [36] designed a pure phase spatial light modulator and computer-controlled diffractive optical element to shape Bessel-like beams. By changing the designed phase profile, the non-diffractive length can be adjusted in specific applications. The team succeeded in manufacturing microholes with lengths ranging from 248 to 904 μm and minimal radius variations (approximately 1.6 μm), achieving a high aspect ratio of up to 560, which is a confirmation of the tunable length characteristic in PMMA microhole fabrication. Additionally, Jenne et al. [131] designed the aberration-corrected Bessel-like beam for the cutting of angled glass surfaces, which is used to obtain high-quality edges.

To meet the demands for beam characteristics in various application fields, researchers are pursuing a variety of more flexible and precise Bessel beam shaping methods. Yu et al. [132] designed a segmented deformable mirror for generating Bessel beams, which functions for both wavefront correction and beam shaping. The result was the production of zero-order and higher-order Bessel beams with adjustable transverse and longitudinal shapes, and the beam quality was significantly improved through phase optimization. In three-dimensional integrated circuit applications, sidelobe suppression is a critical issue. To manufacture high-quality through-silicon vias (TSVs), He et al. used a custom binary phase plate for phase modulation. The generated femtosecond Bessel beam had sidelobes as low as 0.6%, and there was no sidelobe ablation on the 2D array of 10 μm diameter TSVs produced on a 100 μm thick silicon substrate [133]. In ultrafast Bessel laser processing, it is necessary to maintain the energy throughput during Bessel beam propagation. To achieve this goal, Ouadghiri-Idrissi et al. [125] used a non-iterative pure phase spatial light modulator to directly generate high-throughput Bessel beams with arbitrary on-axis intensity shapes in space. Unlike previous approaches that mainly focused on adjusting the cone angle and input pulse energy, they proposed a new perspective the following year [134] to control the longitudinal intensity distribution of Bessel beams for better application in nonlinear processing and plasma manipulation. When high-intensity laser processing is required, Meyer et al. [135] developed an ultrafast beam shaper with an adjustable working distance and no intermediate focus, capable of producing ultrashort Bessel beams with a high cone angle of 23° and a high-intensity focal region with an aspect ratio exceeding 10,000. They also demonstrated its feasibility for stealth cutting of 1cm thick glass. For applications in optical communication and sensing, where long-distance beam propagation is crucial, Zhi et al. [136] recently introduced a new approach. They designed an integrated silicon photonic chip with a unique concentric distributed grating array, capable of generating Bessel–Gaussian beams with a propagation distance of 10.24 m for laser wavelengths ranging from 1500 to 1630 nm, marking a significant breakthrough in extending the propagation distance of Bessel beams.

Regarding processing precision, most research has focused on enhancing lateral resolution. Researchers continue to explore novel methods to further breach resolution barriers. Sun et al. demonstrated the remarkable sub-diffraction-limit resolution of 120 nm using two-photon polymerization to fabricate three-dimensional microbull structures. By forming a highly pure longitudinal light field at the focal point, Li et al. achieved a high aspect ratio in laser-material processing in air with an 800 nm wavelength laser featuring 10 nm characteristic sizes [137]. More recently, Tsuru et al. utilized radially polarized laser beams for high-precision laser micromachining on glass surfaces, achieving micro-ablated pits with a diameter of approximately 67 nm (approximately λ/16) on the opposite side of the glass [138]. These achievements contribute significantly to advancing lateral resolution in laser processing. Nevertheless, it is noted that research on enhancing longitudinal resolution remains scarce, complicated by the combined influence of processing materials, parameters, and environmental conditions, as well as threshold effects. Especially for extreme lateral resolution applications such as laser cutting of millimeter-thick glass, once the lateral processing resolution reaches 100 nm, the aspect ratio sharply drops to 100 or lower, accompanied by longitudinal performance inhomogeneity. 

**Figure 9:**
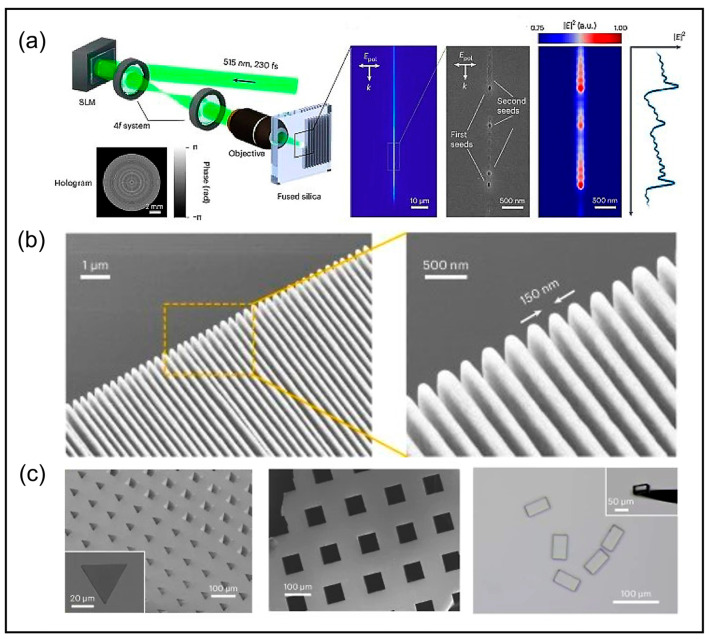
Super-stealth dicing (SSD) by ultrafast laser back-scattered interference crawling [139]. (**a**) Experimental setup for laser processing; (**b**) SEM images and close-up-view of silica nanogratings with a period of 300 nm etched in 2% hydrofluoric acid for 300 s and using 250 μm^−1^ pulse density; (**c**) SEM images of drilling of specific shapes on silica glass etched in 5% hydrofluoric acid for 300 s and optical microscope image of microscale silica cuboids collected after wet etching.

To address this, the recently developed ultra-stealth cutting (SSD) technology in Figure 9a demonstrates the immense potential [139]. The key is the three-dimensional self-organized feedback between far-field and near-field light-matter interactions, enabling nanostructures with lateral resolutions of tens of nanometers and aspect ratios ranging from 1000 to 10,000. They employed a specialized spatial light modulator to stretch the laser beam to dimensions spanning tens of microns while remarkably overcoming the undesirable divergence and non-uniformity issues along the optical axis of the elongated beam. As illustrated in Figure 9b, the cross-sectional profile of silica nanogratings, fabricated through a combined process of modulated laser ablation and subsequent chemical etching with hydrofluoric acid, exhibits excellent longitudinal uniformity, providing tangible evidence for the superiority of SSD technology in high-aspect-ratio nanodicing applications. Furthermore, the tailored shaping capabilities of the SSD technique enable scalable and precise drilling for various shapes, as exemplified in Figure 9c. 

In contrast to filamentation-induced high-index channels through Kerr effect self-focusing, the technology exploits rapidly varying dielectric function distributions to excite optical near-fields, achieving nanoscale light confinement. The newly observed laser-matter interaction mechanism imposes no specific requirements on laser intensity or phase, merely requiring the creation of nanoscale low-index regions. By bypassing the limitations of conventional methods, the innovative approach paves the way for high precision and high-aspect-ratio processing in ultrafast laser manufacturing and processing. 

## 5. Conclusions and Outlook

Ultrafast laser processing with high aspect ratios enables precision, efficiency, and non-contact manufacturing and it has a good application prospect in many fields. In optics, the aspect ratios of optical components such as gratings, lenses, and microlens arrays significantly impact their optical performance. High-aspect-ratio processing facilitates the realization of finer optical structures, enhancing the performance and precision of optical elements [140,141]. In the realm of biological manipulation, high-aspect-ratio processing enables the fabrication of three-dimensional micro-nanostructured biological scaffolds, providing crucial support for cell adhesion, diffusion, and growth, therefore serving as vital tools and platforms for medical research [142,143]. Within microelectronics, nanofabrication via holes with high aspect ratios, facilitated by this technology, is widely adopted in the packaging of three-dimensional integrated circuits [144,145]. The fabrication of high-aspect-ratio structures necessitates the achievement of a substantial disparity between depth and width within extremely minute dimensions, imposing exceedingly high demands on processing precision, material properties, and process stability. Consequently, ultrafast laser processing with high aspect ratios remains a significant challenge in the field of microfabrication.

Filamentation and Bessel-like beam generation are key technologies currently employed for realizing high-aspect-ratio laser processing. Laser filamentation in transparent media stems from the dynamic balance between Kerr effect self-focusing and plasma defocusing. Nonlinear effects confine laser pulse energy within a narrow region, mitigating diffraction-induced lateral divergence, enabling long-distance propagation in media and, consequently, large-depth processing. However, the critical state of filamentation is fragile and prone to disturbances from external factors, posing challenges to the stability and controllability of filamentation-based processing. Bessel beams, with their unique properties, significantly enrich the avenues for ultrafast laser high-aspect-ratio processing. Notably, their non-diffracting nature ensures that the beam spot shape and intensity remain constant over extended propagation distances. The extremely small central spot diameter signifies a high degree of beam energy concentration. The elongated focal region allows for the single-pass fabrication of high-aspect-ratio structures, drastically enhancing processing efficiency. However, generating and manipulating Bessel-like beams tailored to specific applications is challenging, necessitating high-precision optical systems and intricate control strategies, which elevate equipment costs and complexity.

To achieve both high precision and high aspect ratios in processing, future research should delve deeper into laser-material interaction mechanisms, particularly nonlinear ones, explore novel focusing techniques, and optimize beam propagation and energy distribution characteristics.
